# Do Not Give Up Your Stethoscopes Yet—Telemedicine for Chronic Respiratory Diseases in the Era of COVID-19

**DOI:** 10.3390/life12020222

**Published:** 2022-01-31

**Authors:** Stephen Simeone, Daniel Condit, Evan Nadler

**Affiliations:** 1 Department of Internal Medicine, University of Connecticut Health Center, Farmington, CT 06030, USA; ssimeone@uchc.edu; 2 Department of Pulmonology and Critical Care Medicine, University of Connecticut Health Center, Farmington, CT 06030, USA; 3 Department of Pulmonology and Critical Care Medicine, St. Francis Hospital and Medical Center, Hartford, CT 06105, USA

**Keywords:** telemedicine, teleconsultation, chronic obstructive pulmonary disease, asthma, COVID-19, intensive care unit

## Abstract

Telemedicine in its many forms has been utilized across numerous medical specialties to facilitate and expand access to medical care, optimize existing healthcare infrastructure to encourage patient–provider communication, reduce provider burnout, and improve patient surveillance. Since the emergence of the novel coronavirus (COVID-19) pandemic there has been widening of existing socioeconomic disparities in healthcare access for those with chronic respiratory diseases, sparking interest in expanding the use of telemedicine modalities to enhance access to pulmonology specialist care, pulmonary rehabilitation, symptom monitoring, and early identification of clinical exacerbations. Furthermore, the use of telemedicine has been expanded into the intensive care setting to improve patient outcomes and offset provider demands following the increase in critically ill patients due to COVID-19. While an invaluable modality by which to broaden healthcare access and increase the efficacy of care delivery, telemedicine must be used in conjunction with face-to-face physical evaluation and appropriate clinical testing to optimize its benefit. We present here our view of the benefits and disadvantages of the use of telemedicine in the management of chronic respiratory disorders from the perspective of practicing clinicians.

## 1. Introduction

Telemedicine, or the use of audio, text, and visual telecommunication technologies to de-liver remote direct medical care to patients and enhance communication between providers, has become more widespread in the past two decades. The use of telecommunications technology to broaden access to healthcare found its origins during the American Civil war after which the introduction of the telephone at the turn of the twentieth century was readily employed by physicians [1]. The middle of the twentieth century brought techno-logical advancement facilitating faster and more detailed communication and patient monitoring as well as a broadening of the applications for telecommunications in medical care. During the emergence of space exploration in the 1960s, the National Aeronautics and Space Association utilized telecommunications technology to remotely monitor the clinical parameters such as heart and respiratory rate of astronauts to characterize the physiologic effects of zero gravity [1]. Given the broad range of telecommunication technologies and platforms available, telemedicine can be sub-classified into different categories based on its specific application to a patient’s care needs. Telemonitoring refers to the use of telecommunications technology equipped to record and transmit patients’ vital signs and symptoms reports to providers remotely, while telemedicine consultation refers to patient-physician encounters which occur remotely over an audio-visual interface [2]. Telerehabilitation refers to the rehabilitation services which are mediated over audio-visual interfaces rather than occurring in person at a facility [3]. This has led, often by necessity, to the widespread use of telemedicine interventions, some of which may not have high quality research supporting their effectiveness, cost-benefit, or safety. Indeed, sound clinical care is by no means defunct but rather needs to be the conduit through which these novel technologies are introduced and applied.

Prior to the novel coronavirus (COVID-19) pandemic, telemedicine was deployed to in-crease accessibility across healthcare systems and improve the quality of care provided to remote, underserved, and impoverished communities [4,5]. The frequency of telemedicine encounters has increased significantly in the past two decades, with the average annual compound growth rate increasing 52% annually between 2005 and 2014 and 261% from 2015 and 2017 [6]. Concerns regarding disease transmission in the setting of COVID-19 sparked a renewed interest in telemedicine, with the Centers of Disease Control and Prevention (CDC) reporting a 50% increase in telehealth visits in the first quarter of 2020, compared with the same period in 2019 [7]. Furthermore, there was a 154% increase in visits noted in surveillance week 13 in 2020 [7]. In one analysis of telemedicine use at New York University Langone Health, there was a 683% increase in urgent care telemedicine visits between March and April of 2020 attributed to the then emerging COVID-19 pan-demic [8]. Given the increased patient burden shouldered by the healthcare system in the face of the COVID-19 pandemic, there is increasing interest in shifting provider-patient encounters out of the clinic and into our patients’ homes to minimize unnecessary face-to-face contact and reduce transmission of the novel coronavirus [9]. 

Within this perspective article, we hope to provide a clinician’s perspective on the benefits and limitations of telemedicine in the treatment of respiratory diseases in the inpatient, outpatient, and critical care settings. Our goal is to provide an adequate perspective based on the experiences of the many clinicians involved in the day-to-day clinical management of respiratory patients and discuss these observations in the broader context of the published literature. We also discuss the increased use and broadening of applications of telemedicine following the emergence of the COVID-19 pandemic.

## 2. Materials and Methods

### 2.1. Literature Resources

With the intention of framing our clinical perspective in the broader context of the published literature, a literature review was undertaken using the Google Scholar and PubMed databases and used the search terms “telemedicine”, “telemonitoring”, “history of telemedicine”, “telemedicine and critical care”, “telemedicine and intensive care medicine”, “telemedicine and asthma”, “telemedicine and chronic obstructive pulmonary disease”, “telemedicine and dyspnea”, “telemedicine and pulmonary nodules”, “telemedicine and cost”, “telemedicine and COVID-19”, and “telemedicine and physical exam”. 

### 2.2. Selection and Exclusion Procedures 

All investigations were screened by two reviewers (SS and DC). A total of 111 primary investigation, review, or perspective articles were evaluated and 58 were included in this commentary article based on their relevance to the discussion on the utility of telemedicine in the diagnosis, treatment, and management of respiratory disease in the United States, methodologic comprehensiveness, sample size of evaluation (if applicable), and recentness of publication. Primary research investigations were excluded if their methodology was unclear or absent and review articles were excluded if they did not address a topic germane to the main foci of this commentary article. 

## 3. Telemedicine Prior to the Emergence of COVID-19

Prior to the emergence of the COVID-19 pandemic, telemedicine had been deployed as an alternative and complementary medium to facilitate diagnostic evaluation, patient monitoring, and counseling across a broad range of clinical environments (see Table 1). In ambulatory primary care medicine, telemedicine was effectively utilized to increase remote symptom monitoring and access to clinical counseling with particular focus in higher risk patient demographics. Orozxo-Beltran et al. [10] demonstrated that use of a virtual telecommunication platform enabling early symptom reporting led to a significant reduction in disease exacerbations, emergency department use and hospital admissions in patients with high-risk chronic diseases including diabetes mellitus, congestive heart failure and chronic obstructive pulmonary disease (COPD). Müller et al. [11] assessed the efficacy and all cause complication rate of non-acute headache assessment using in-person clinical evaluation versus telemedicine-enabled evaluation and found no significant difference in the rate of complications at 12 months follow up. Benefits of telemedicine in critical care medicine have also been shown; a large meta-analysis of 11 observational investigations by Wilcox and Adhikari [12] concluded that the application of telemedicine was associated with a significant reduction in in-hospital mortality. Furthermore, a non-randomized large-scale analysis of 118,990 intensive care patients by Lilly et al. [13] similarly concluded that telemedicine, when deployed for frequent audio, visual, and vital sign monitoring, led to reductions in adjusted length of stay and in-hospital mortality. In the field of urology, telemedicine has been deployed to not only reduce risk of transmission and personal-protective equipment but has led some to suggest that many specialists could remotely undertake consultations, decreasing waiting times and increasing access to specialty providers [14]. The role of telemedicine in respiratory disorders of all acuities has become of particular interest as, like many fields of medicine, their optimal diagnosis and management requires a synthesis of comprehensive patient history, physical exam, serology, radiography, and symptom monitoring.

## 4. Telemedicine in Outpatient Management of Chronic Respiratory Diseases

There has been a growing interest in defining the role of telemedicine in chronic respiratory illnesses such as COPD and asthma given their associated morbidity and mortality. While numerous investigations have been undertaken into the broad topic of respiratory disorders, the majority have concerned COPD and asthma given their high prevalence and disease burden. Some investigations have demonstrated a beneficial role for the out-patient management of COPD and other chronic respiratory diseases in rural areas via teleconsultation. Raza et al. [15] undertook an analysis of 314 patients managed for COPD, abnormal thoracic radiology, and dyspnea via teleconsultation in rural Missouri. Not only did they show improved access to subspecialty care, but also that teleconsultation was more cost effective than in person specialty consultation ($313/patient versus $1166 respectively) [15]. Additionally, a randomized-controlled analysis of the role of teleconsultation and telemonitoring for early recognition of exacerbation in COPD demonstrated no significant difference in all-cause mortality, hospitalization frequency, or hospitalization duration at 24 months follow-up between the telemedicine and face-to-face patient cohorts [16]. Bourne et al. [17] demonstrated that online pulmonary rehabilitation was non-inferior to conventional facility pulmonary rehabilitation when modified Medical Research Council (mMRC) scores and 6-minute walk tests were compared among participants following completion of six-week programs. A subsequent analysis by Vasilopoulou et al. [18] similarly demonstrated non-inferiority in telemedicine enabled home-based (HB) pulmonary rehabilitation and further demonstrated that compliance with only HB pulmonary rehabilitation, rather than facility based, was an independently associated with reductions in emergency department usage. Furthermore, a meta-analysis by Liu et al. [19] demonstrated that despite heterogeneity in specific telemedicine implementation practices, telemedicine integration in the management of COPD lead to decrease hospitalization rates (OR: 0.24, CI: 0.20-29). While these analyses certainly suggest a clear benefit of telemedicine in the clinical maintenance management of chronic respiratory disease, several recent me-ta-analyses suggest that additional investigation is warranted [20].

Similar evidence exists for the burgeoning role of telemedicine in the management of asthma; Portnoy et al. [21] demonstrated that comprehensive teleconsultation and tele-monitoring of pediatric asthma patients using digital stethoscopes and otoscopes was non-inferior to face-to-face evaluation. Of growing consideration is the integration of tele-medicine into school environments to increase patient access to specialty care and facilitate closer symptom monitoring in pediatric populations. A randomized-controlled investigation integrating teleconsultation in addition to supervised therapy in schools for pediatric asthma management demonstrated significantly increased asthma symptom-free days (11.6 vs. 10.97, *p* = 0.01) and reduced emergency department use or hospitalizations (OR: 0.52 CI: 0.32-0.84) when compared to supervised therapy only consultation [22]. Notably, teleconsultations in this analysis were undertaken in conjunction with face-to-face interactions with school nurses trained to administer medications to ensure medication compliance. Another more recent large-scale retrospective analysis by Bian et al. [23] demonstrated a 21% relative decrease in emergency department use for asthma patients following school-based teleconsultation in a rural community over three years. The largest reduction in emergency department presentations (35%) occurred in the final year of the investigation suggesting that telemedicine initiatives may require a roll out period before their maximal benefits can be accurately assessed [23,24]. To address this initial apprehension among patients due to unfamiliarity with the process of navigating an unfamiliar telemedicine platform, other analyses have integrated comprehensive telemedicine management with smartphone-based platforms. Mammen et al. [25] introduced a multifaceted telemedicine platform for remote asthma management composed of smart-phone based teleconsultation with nurses, remote symptom tracking, and EMR based software among 33 adult patients for six months and demonstrated a 39.71% increase in medication adherence and 43.4% increased guideline compliance among providers. However, in a systematic review of school-based telemedicine initiatives, Kim et al. [26] concluded that the benefit of these initiatives remains unclear due to a dearth of high-quality evidence. De-spite continued need for more investigations substantiating the role of telemedicine in the outpatient management of asthma, some investigators suggest that telemedicine will pro-vide an irreplaceable medium to monitor patients and tailor their asthma care in the era of COVID-19. Jain et al. [27] assert that video teleconsultation used in lieu of face-to-face encounters was effective in providing instruction on inhaler technique, assessing compliance, and altering treatment plans in 22.4% of asthma patients who had unreliable follow up due to COVID-19.

## 5. Telemedicine in the Management of Critically Ill Patients

The utility of telemedicine to facilitate early recognition of acute medical events and expedite treatment in the critically ill has been evaluated for decades (see Table 2). Following its introduction in the intensive care unit (ICU) in the 1980s, telemedicine modalities have been used to facilitate consultation with intensivist physicians, monitor vital signs and serology, promptly respond to alarms, and update patients’ families [28]. Lilly et al. [13], in their prospective analysis demonstrated a comprehensive telemedicine platform involving real-time audio-visual patient monitoring and EMR based software to detect hemodynamic aberrations led to decreased adjusted ICU mortality (HR: 0.74, *p* ≤ 0.001) and adjusted ICU length of stay (LOS) by up to 4.5 days depending on the patients’ ICU admission length. The telemedicine team providing care in this analysis had complete autonomy over the care decisions made for their patients differing from previous investigations where telemedicine was deployed to increase access to intensivist physicians via teleconsultation [29]. Another investigation by Willmitch et al. [30] undertook an analysis of ICU LOS and all-cause mortality annually three years following the introduction of telemedicine in ten ICUs. They demonstrated significant reductions in both outcomes with the absolute reduction increasing each year, suggesting that the optimal benefit of telemedicine introduction may not manifest immediately following the introduction of a telemedicine service into a healthcare system similar to the phenomenon of delayed benefit identified in the deployment of telemedicine in schools for asthma management by Bian et al. [23,30]. Several investigations have also postulated that improvements to patient outcomes occur because telemedicine can be used to bridge the gap between “open ICUs”, where admitting providers treat critically ill patients with or without the input of a designated intensivist, and “closed ICUs” where critically ill patients are treated solely by intensivist led medical teams [28,31]. While the initial application of telemedicine in the ICU was to enable and expedite intensivist teleconsultation, technological advances have facilitated continuous patient monitoring by remote intensivist teams. A seminal investigation by Rosenfeld et al. [32] demonstrated a 46–68% decrease in adjusted ICU mortality, 44–50% decrease in ICU complications, and 30–34% reduction in ICU LOS following the introduction of remote continuous intensivist monitoring in a previously open ICU. Not only did this investigation demonstrate the beneficial role of remote real time monitoring, but also introduced a model for integrating audiovisual patient assessment, radiology, serology, and comprehensive flowsheet data [32]. In an analysis of this investigation by Celi et al. [28], these profound reductions in mortality, complication rate, and associated cost of admission were in part driven by early identification and management of “outlier patients” whose longer admission lengths increased their risk of complications and adverse outcomes. Summarily, the emerging multifaceted model of telemedicine in the ICU not only optimizes patient access to intensive care physicians equipped with real time clinical information but also facilitates the identification of patients most at risk to develop poor outcomes. 

Given the high medical complexity of critically ill patients and the resultant increased opportunity for medical errors and adverse events, the introduction of telemedicine in ICUs is also postulated to address the deleterious outcomes that occur due to increasing nursing workload. Tarnow-Mordi et al. [33] demonstrated that increased ICU nursing workload was associated with a twofold increase in adjusted all-cause mortality while another analysis by Amaravadi et al. [34] demonstrated a 39% increase in median LOS and 32% increase in admission costs with reduced nurse-to-patient ratios. These challenges were significantly exacerbated by the COVID-19 pandemic with Lasater et al. [35] reporting an increased ratio of patients per nurse (3.3-9.7:1) and concomitant increase in patient dissatisfaction with their healthcare experience following the pandemic’s emergence. An investigation by Kleinpell et al. [36] demonstrated that the introduction of telemedicine begins to address the challenge of increased ICU nursing workload with 63% of nurses reporting telemedicine allows for improved efficiency in completing tasks and 63.6% reporting an improvement in overall job performance. Further yet, a meta-analysis of acceptance of integrating telemedicine into care delivery in the ICU among nursing staff found that 82% of respondents in 23 studies felt that telemedicine yielded improved outcomes in patient care [37]. 

In addition to changing nature of the patient-provider interaction due to the widespread adoption of telemedicine practices for the management of other respiratory diseases, the diagnosis and management of COVID-19 pneumonia and its long-term sequelae has also relied on telemedicine as the pandemic has continued. Many medical systems have expanded use of telemedicine modalities since the emergence of the pandemic with Mann et al. [8] reporting that COVID-19 related teleconsultations exceeded face-to-face encounters during their investigation period with over half of their 683% increase in urgent care virtual visits being COVID-19 related. Additionally, Hamm et al. [38] reported that various telemedicine modalities were used throughout the delivery of care during the COVID-19 pandemic with application in the triaging of patients, supervision of resident physicians and advanced practice providers, and to facilitate medical specialty consultation, all while reducing risk of bidirectional transmission and use of PPE. Telemedicine has also been used to quickly identify symptomatic healthcare workers who may require quarantine. Zhang et al. [39] described the successful deployment of web-based symptom reporting application for healthcare workers which was used by 80-90% of employees and identified over 500 unique cases of symptomatic healthcare workers requiring further clinical assessment. A review by Lukas et al. [40] proposes that telemedicine modalities may also play a critical role in enabling continuous remote vital sign and biomarker monitoring to recognize early clinical deterioration and thus enable the identification of patients requiring escalation of care. Jeong et al. [41] proposed that continuous symptom monitoring with widely available wearable technologies may be able to detect vital sign aberrations such as fever and tachycardia which can not only identify early illness but also aid in the prognostication of patients. However, the authors note that not all consumer-grade wearable or smartphone-based technology offers temperature, heart rate, respiratory rate, and pulse oximetry monitoring and that many are currently approved by the United States Food and Drug Administration for clinical monitoring [41].

## 6. Telemedicine in the Management of COVID-19

The deployment of telemedicine for the diagnosis of COVID-19 has also been challenged by the clinical heterogeneity of the disease and logistical and economic barriers of effective administration. Adans-Dester et al. [42] suggest that clinical syndrome of COVID-19 varies significantly between patients and can manifest with both pulmonary and numerous non-specific extrapulmonary symptoms with fever (85.6%), cough (68.7), and diarrhea (3.7%) predominating the syndrome in another analysis; shortness of breath (18.6), pharyngodynia (13.6), and nasal congestion (4.8%) were comparatively rare components of the clinical presentation in this analysis [43]. This clinical heterogeneity and non-specific nature of initial symptomatology represents a logistical challenge in differentiating COVID-19 from other upper respiratory infections and thus requires that a large number of patients be monitored if they are under suspicion for the disease. Further challenging still is the prospect of monitoring asymptomatic transmission; an investigation of a COVID-19 outbreak in a skilled nursing facility by Arons et al. [44] demonstrated that 56% of residents who tested positive reported no symptomatology and a further 8% reporting only atypical symptoms such as nausea, vomiting, dizziness, or chills. In summary, while telemedicine has been deployed since the emergence of the COVID-19 pandemic to facilitate early diagnosis while minimizing transmission risk and monitor healthcare providers symptomatology, more investigation is needed to further optimize its potential benefits in fighting the COVID-19 pandemic. 

## 7. Limitations of Telemedicine in Managing Chronic Respiratory Diseases

While telemedicine has demonstrated advantages in providing remote clinical management and monitoring of patients with chronic respiratory diseases, determining where in the course of clinical diagnosis, treatment, and monitoring to utilize telemedicine remains an area of debate. Some investigations have demonstrated benefits following the employment of telemedicine while others suggest these benefits may not extend to all patient demographics. In the treatment of COPD, Berkhof et al. [45] compared several quality-of-life indices (Clinical COPD Questionnaire) between patients monitored via telemedicine and conventional office-based encounters and demonstrated that telemedicine was associated with significantly lower CCQ scores, and significantly higher pulmonology follow up appointments. Furthermore, there is evidence that increasing accessibility to healthcare providers via telemedicine does not lead to improved outcomes. Pinnock et al. [46] demonstrated that telemonitoring of COPD patients led to no significant difference in time to hospital admission, duration of admissions, or quality of life indices despite a 28.7% increase in provider contacts (telephone contacts and home visits). 

The inconsistencies between investigations characterizing the benefit of telemedicine are attributable to how telemedicine has been paired with face-to-face encounters and in-person physical examinations. In the analysis by Raza et al. [15] of patients assessed in a rural community by teleconsultation, 90% of patients underwent a physical examination by a nurse as part of their clinical evaluation. Similarly, in the analysis by Portnoy et al. [21] demonstrating the non-inferiority of teleconsultation in the management of pediatric asthma, the virtual consultation and physical assessment enabled by digital stethoscope and otoscope was augmented by face-to-face encounters for asthma education, spirometry, and venipuncture. Of additional consideration is the parity between conventional face-to-face physical evaluations and the virtual physical examinations incorporated into teleconsultation and telemonitoring. In a prospective concordance investigation by Akhtar et al. [47] there was poor agreement between face-to-face physical evaluation and teleconsultation examination, suggesting that there is utility in incorporating face-to-face physical examination into telemedicine examinations to ensure that patients are ensured the greatest benefit from the increased access to subspecialty care which telemedicine affords. Some investigations, such as that of Pacht et al. [48], have also considered the role of electronic stethoscopes to enable providers to perform real time virtual pulmonary examinations and have demonstrated encouraging agreement between face-to-face auscultation and the virtual examination [49]. However, the wide-scale application of this technology is currently limited by cost and the logistics of deploying this technology into communities with fever resources. 

Several investigations have ascertained reduced mortality and LOS following introduction of telemedicine technology in the ICU while other findings suggest that these benefits are confounded by the logistics and cost of incorporating telemedicine into a healthcare system. Kumar et al. [50] considered the introduction of various telemedicine modalities in ICUs from 1990 to 2011 and demonstrated not only significant cost of introduction ranging from $50,000 to $100,000 per ICU bed but a variable effect on cost effectiveness once introduced; while some analyses demonstrated reductions in the cost of care of up to $3000, others demonstrated an increase of up to $5600 per patient. Another more recent investigation also demonstrated increased LOS (7.3 vs. 6.8 days) and mean admission cost ($13,180 vs. $12,301) of patients treated with and without telemedicine consultations respectively; however, hospital mortality was significantly improved in the telemedicine consultation cohort (4.4% vs. 5.2%, *p* = 0.013) [51].

While telemedicine has certainly provided a novel toolset by which to increase access to underserved patient populations, the logistics of employing telemedicine practices in these communities remains a complex challenge. Drake et al. [52] considered the dynamics of broadband internet access and physician accessibility in the rural and urban communities across the United States and concluded that broadband penetration rates in rural communities were considerably reduced versus those of urban communities (82.7% vs. 96%). Furthermore, in analyzing a subset of communities of approximately 4 million patients with significantly reduced broadband access (deemed “communities with extreme access considerations”), broadband penetration decreased to 59.9%. Nouri et al. [53] also discussed disparities in technology ownership in impoverished populations, reporting that only 71% of impoverished patients own a smartphone and only 53% have basic digital literacy. Of equal consideration to telecommunications infrastructure is the technological literacy requisite to effectively use the telemedicine technology available. Following expansion of telemedicine technologies at Columbia University Irvine Medical Center, Ye et al. [54] undertook an electronic medical record (EMR) mediated analysis of usage patterns among their patient cohort and concluded that age ≥ 45 years, African American or Hispanic race, and Medicare/Medicaid enrollment were significantly associated with decreased use of telemedicine technology. Furthermore, other recent investigations characterizing the relationship between patient demographics and digital literacy by Boriani et al. [55] and Hsaio et al. [56] demonstrated that increased age, uninsured status, living in a rural community, and reduced education level were also significantly associated with non-use of internet-based platforms. Summarily, the reduced telecommunications infrastructure in combination with reduced digital literacy and technology ownership continues to compose the “digital divide” between healthcare providers and these underserved patient populations.

## 8. Discussion

Telemedicine has undoubtedly made a positive contribution to healthcare accessibility amidst the challenge of healthcare delivery during the COVID-19 pandemic. However, the growing adoption of telemedicine has once again re-ignited the discussion as to the value of the physical examination in the clinical encounter. In a meditation on the changing role of the physical examination following the emergence of the pandemic, Dr. Paul Hyman has suggested that the physical examination not only forms an objective basis by which to ground the clinical encounter and build the medical narrative but is indispensable in cultivating the human connection that forms the foundation of the patient-practitioner relationship [57]. Clinical interactions via phone or video, while beneficial in enabling social distancing and increasing access to healthcare providers, are unlikely to replace patients and their physicians positioned directly across from one another. Zuiderent et al. [58] further suggest that the environment context (e.g., clinic) and components of the clinical encounter such as the physical examination constitute the situational environment need-ed for healthcare providers to untangle their patients’ stories and formulate an appropriate plan of care; reduction of the clinical interaction to a virtual encounter reduces its “context density” such that quality and nature of the communication between patient and provider changes. While innumerable in its applications, the body of evidence suggests that telemedicine is most effective when used synergistically with face-to-face assessment and physical evaluation to optimize patient involvement while ensuring the most thorough clinical evaluation to properly diagnose and treat patients.

## 9. Conclusions

The management of respiratory disorders of all acuity requires a balance of unencumbered communication with our patients to gather the story of their illness, effective physical evaluation, thoughtful diagnostic testing, and careful synthesis of these factors to best inform their care. Telemedicine is a powerful tool for reducing the distance between our patients and the clinic and facilitating communication but must be used alongside the other clinical tools available to modern providers to deliver well-informed medical care.

## Figures and Tables

**Table 1:** Applications of telemedicine in the diagnosis, management, and monitoring of respiratory diseases.

Component of Healthcare	Details
Diagnosis and Triaging	-Enable patient–provider encounters while reducing risk of infectious disease. -Increase access to evaluation by physicians and advanced practice providers via teleconsultation. -Enable expedient triaging of patients in urgent care and emergency departments via teleconsultation. -Expedite access to specialty medical care via remote teleconsultation from a centralized telemedicine center. -Identify patients who require additional evaluation based on reported symptoms.
Treatment	-Facilitate access to providers to discuss medication efficacy or changes in symptoms which may warrant further diagnostic evaluation or changes in course of care. -Reduce nursing workload by increasing access to the care team. -Encourage medication compliance and participation in rehabilitation treatments.
Monitoring	-Monitor vital signs and serology to identify clinical deterioration.

**Table 2:** Summary of Investigations of the utility of telemedicine in the intensive care unit.

Investigation	Sample Size	Selected Primary Findings
Lilly et al. [13]	118,990	*-Significant adjusted hospital (HR: 0.84, CI: 0.78-0.89) and intensive care unit (HR: 0.74, CI: 0.68-0.79) mortality reduction for telemedicine cohort. -Adjusted length of stay by 0.5, 1.0, and 3.6 days for patients who stayed in the hospital for ≥7, ≥14, and ≥30 days respectively. -Adjusted intensive care unit length of stay by 1.1, 2.5, and 4.5 days for patients who stayed in the intensive care unit for ≥7, ≥14, and ≥30 days respectively.*
Willmitch et al. [30]	24,656	*-Severity adjusted hospital length of stay was significantly reduced by 14.2% and severity adjusted intensive care unit length of stay was reduced by 12.6% following introduction of continuous remote intensivist monitoring. -Relative risk of in-hospital mortality was also significantly reduced by 23%.*
Rosenfeld et al. [32]	225 (Period 1) 202 (Period 2) 201 (Intervention)	*-Severity adjusted intensive care unit mortality decreased by 46-68% and severity adjusted hospital mortality decreased by 30–33% following by introduction of continuous remote intensivist monitoring. -Intensive care unit length of stay also decreased by 30–34% and intensive care unit admission cost decreased by 33–36%.*

## Data Availability

Not applicable.
